# Utilization of the Ballast Long Guiding Sheath for Neuroendovascular Procedures: Institutional Experience in 68 Cases

**DOI:** 10.3389/fneur.2021.578446

**Published:** 2021-05-07

**Authors:** Ameer E. Hassan, Elizabeth M. Burke, Marlon Monayao, Wondwossen G. Tekle

**Affiliations:** ^1^Department of Neurology, University of Texas Rio Grande Valley, Harlingen, TX, United States; ^2^Department of Neuroscience, Valley Baptist Neuroscience Institute, Harlingen, TX, United States; ^3^Department of Biological Sciences, The Ohio State University, Columbus, OH, United States

**Keywords:** endovascular therapy, aneurysm, stroke, stent, long guiding sheath

## Abstract

**Background:** The rise of neurointerventional devices has created a demand for guide systems capable of navigating to the carotid artery consistently regardless of tortuosity. The shift toward large distal access catheters (DACs) and desire for greater trackability have inspired the creation of flexible, supportive, large-lumen long guiding sheaths. Recently, the Ballast long guiding sheath was introduced to provide stability and flexibility while navigating neurointerventional devices through tortuous intracranial anatomy.

**Objective:** To report our experience using the Ballast long guiding sheath in a series of patients undergoing a variety of neuroendovascular procedures.

**Methods:** We retrospectively identified all patients who underwent neuroendovascular treatment with a long guiding sheath were selected from a prospectively maintained endovascular database from January 2019 to November 2019. Baseline clinical characteristics and procedural details were collected.

**Results:** A total of 68 patients were included, mean patient age 67.6 ± 13.6 years. Of the patients treated, 52.9% (36/68) presented with stenosis, 25% (17/68) with aneurysms, 13.2% (9/68) with stroke or emboli, 1.5% (1/68) with a tumor, 1.5% (1/68) with an arteriovenous fistula (AVF), and 4.4% (3/68) with a carotid web. Of the patients with stenosis, 20/36 (55.6%) were extracranial, and 16/36 (44.4%) were intracranial. The Ballast long guiding sheath was used to deliver treatment devices for stenting (37/68, 54.4%), flow diversion (12/68, 17.6%), mechanical thrombectomy (8/68, 11.8%), endovascular coiling (5/68, 7.4%), liquid embolization (3/68, 4.4%), balloon angioplasty (2/68, 2.9%), and balloon angioplasty with stenting (1/68, 1.5%). No Ballast-related complications or adverse events were encountered.

**Conclusions:** We demonstrate the feasibility of the Ballast long guiding sheath to successfully deliver modern neurointerventional treatment devices through tortuous anatomy.

## Introduction

As neuroendovascular intervention expands to new applications, and especially as the devices used—from large-bore distal access catheters (DACs) (1) to a wide range of flow diverters (2, 3)—expand in both size and variety, it is increasingly important for long guiding sheaths to be useable through multiple access options, for a range of procedures, across lesion locations. Delivery of large devices to the target location in the cerebrovasculature necessitates large inner diameters (ID) to be paired with trackability, especially under emergent conditions (4) or through tortuous anatomies (5).

Furthermore, while transfemoral access has been the preferred approach during neurointerventional procedures for decades (6), recent studies have shown the promise of transradial access for the diagnosis and treatment of various cerebral pathologies with embolization, mechanical thrombectomy, microsurgical aneurysm clipping, stenting, and balloon occlusion tests (7–9). While studies of safety and efficacy of radial access are ongoing, a key and understudied aspect of this procedure is the feasibility of using current long guiding sheaths in this novel application across disease states.

Long guiding sheaths are designed to provide stability while advancing neurointerventional devices across the aortic arch and distal flexibility for advancement in the carotid artery, but analysis concerning the performance of guide technologies is generally published only as subsidiary information to patient outcomes from the treatment device (10). Furthermore, there is scant published evidence concerning device performance in both transradial and transfemoral applications across a range of neuroendovascular procedures (11). In this report, we describe our experience with the Ballast 088 long guiding sheath (Balt USA, LLC) in diagnostic angiography as well as across the treatment of various cerebrovascular diseases, including aneurysms, acute ischemic stroke, intracranial and extracranial stenosis, arteriovenous fistula (AVF), carotid webs, and tumors.

## Materials and Methods

### Ethics

The studies involving human participants were reviewed and approved by the MetroWest institutional review board. The patients provided their written informed consent to participate in this study.

### Patient Selection

All patients who underwent neuroendovascular treatment with a Balt Ballast long guiding sheath were selected from a prospectively maintained endovascular database at Valley Baptist Medical Center from January 2019 to November 2019.

### Data Collection

Baseline clinical characteristics were recorded, including demographic information, comorbidities, medication usage, lesion characteristics, and target vessel location. Procedural characteristics of interest included procedure type, fluoroscopy time, operative room time, safety outcome rates, and patient outcomes were gathered from the selected patients. Safety outcome data included incidence of dissection, iatrogenic stroke, or intraprocedural rupture.

### Endovascular Procedures

All embolization procedures were performed under general anesthesia, while mechanical thrombectomy and carotid stenting were performed under conscious sedation. Systemic anticoagulation with heparin, radial cocktail, and/or Aggrastat, were given during the procedure in the majority of cases.

#### Mechanical Thrombectomy

A Solumbra approach (12) was used in which a Ballast.088 long guiding sheath, a DAC, and microcatheter were used to cross the lesion. The Ballast was navigated into the vertical petrous or proximal V3 vertebral segments. A stent retriever was then deployed for 5–7 min and pulled back under aspiration along with the DAC. Heparin was not administered in any of our stroke cases; however, 2,000 units/bag are connected to flush lines. GP2b/3a inhibitors were not routinely used. The procedure was typically terminated after 60 min of intervention time. The approach for radial cases utilizes a short 6 Fr Glidesheath Slender (Terumo Interventional Systems). A radial cocktail (2.5 mg verapamil, 2,000 U heparin, 200 mcg nitroglycerin) was then infused to prevent radial spasms, and a roadmap image was obtained to determine the size of the radial artery. Next, a 5 Fr Simmons/Sidewinder Slender (Terumo Interventional Systems) was advanced over a baby J wire. A stiff 035 exchange length wire was then advanced to exchange the Sidewinder and remove the 6 Fr sheath. Finally, the Ballast long guiding sheath was navigated over its dilator to the vessel of interest, and the procedure was performed.

#### Carotid Stenting

The common carotid artery (CCA) was cannulated and mapped under fluoroscopic guidance with a triaxial catheter system and Ballast long guiding sheath. The guide wire and catheter were then advanced into the distal CCA. A distal embolic protection device was advanced into the distal cervical segment of the internal carotid artery and the device deployed and the delivery system withdrawn. A small balloon was typically advanced to pre-dilate the lesion and a stent was deployed and then we post-dilate and retrieve the distal embolic protection device. If results were satisfactory, the catheters and sheaths were removed and a closure device was used to close the arteriotomy site.

#### Liquid Embolization

Liquid embolic agents were slowly introduced under fluoroscopic control via a triaxial catheter system with the Ballast long guiding sheath. Once in contact with the patient's blood, the agent precipitated into an embolus *in situ* and the catheters were removed. The duration of the injection was ~ 30 min.

#### Flow Diversion

Flow diverters were deployed through a microcatheter into the parent artery by using a triaxial guide-catheter system with the Ballast long guiding sheath. Fluoroscopy was used to ensure correct apposition of the flow diverter, with additional CT angiography used at the surgeon's discretion. If there were no evident complications, the delivery catheter was removed and the procedure was completed.

#### Endovascular Coiling

A detachable platinum coil was inserted into the aneurysm with fluoroscopic guidance using a triaxial catheter system with a Ballast long guiding sheath for proximal support. Once within the aneurysm, the coil was separated from the catheter and left to occlude blood flow to the aneurysm. If necessary, multiple coils were used.

#### Balloon Angioplasty

A balloon guide catheter was advanced through a Ballast long guiding sheath into a blood vessel with fluoroscopic guidance until correctly positioned. The balloon was then inflated at the blockage site for 30–60 s intervals at 4–6 atm until recanalization was obtained. Intra-arterial r-tPA was added once distal embolization was observed. The procedure was terminated and the introducer sheath was removed once flow through the vessel was sufficiently restored.

### Statistical Analysis

All statistics of a given metric were computed on the set of patients for which the metric was reported. Categorical data are presented as *n/N* (%), by category. Continuous data are presented as mean ± standard deviation (SD) for symmetrical distributions, else median ± interquartile range (IQR). Numerical data are presented as mean ± standard deviation (SD), or *n* (%). All computations were performed using the R programming language (13).

## Results

### Patient Characteristics

A total of 68 patients were included in the study, including 34 (50.0%) females and 34 (50.0%) males. Patient baseline and lesion characteristics are presented in Table 1. Mean patient age was 67.6±13.6 years. Of the patients treated, 52.9% (36/68) presented with stenosis, 25% (17/68) presented with aneurysms, 13.2% (9/68) presented with stroke or emboli, 1.5% (1/68) was treated for a tumor, 1.5% (1/68) for an AVF, and 4.4% (3/68) were treated for a carotid web. Of the patients treated with stenosis, 20/36 (55.6%) of the stenoses were extracranial and 16/36 (44.4%) were intracranial. The extracranial stenoses were located in the right (10/19, 52.6%) and left (9/19, 47.4%) internal carotid artery (ICA). The intracranial stenoses were located in the right supraclinoid (1/16, 6.25%), left supraclinoid (2/16, 12.5%), left middle cerebral artery (MCA) M1 or M2 segment (4/16, 25%), right MCA M1 segment (2/16, 12.5%), left vertebral artery (VA) (1/16, 6.25%), right VA (2/16, 12.5%), basilar artery (BA) (1/16, 6.25%), right pre-cavernous segment of the ICA (1/16, 6.25%), and right posterior cerebral artery (PCA) P1-P2 segments (2/16, 12.5%). All intracranial stenosis patients were symptomatic and failed medical therapy at least once. These patients were treated with a gateway balloon and typically underwent stenting with the Wingspan Stent System (Stryker). Overall, 10.6% of the cases were previously treated for their condition. Lesions were most commonly located in the anterior circulation (54/66, 81.8%), with the remaining lesions in the posterior circulation (12/66, 18.2%). Lesion locations included the ICA (26/67, 38.8%), the MCA (15/67, 22.4%), the VA (4/67, 5.97%), and the CCA (1/67, 1.49%).

**Table 1:** Patient and lesion characteristics.

**Characteristic**	**Frequency (*N* = 73)**
Age, mean ± SD	67.6 ± 13.6
Female (%)	34/68 (50%)
Comorbidities	
Congestive heart failure	2/68 (2.9%)
Hypertension	55/68 (81%)
Chronic obstructive pulmonary disease	3/68 (4.4%)
Coronary artery disease	5/68 (7.4%)
Diabetes mellitus	37/68 (54.4%)
Peripheral vascular disease	3/68 (4.4%)
Atrial fibrillation	5/68 (7.4%)
Renal failure	7/68 (10.3%)
Hypothyroidism	1/68 (1.5%)
History of SAH	1/68 (1.5%)
History of ICH	0/68 (0%)
History of aneurysm	2/68 (2.9%)
Hypersensitivity lung disease	17/68 (25%)
History of stroke	23/68 (33.8%)
Hypercholesterolemia	7/68 (10.3%)
Lesion type	
Stenosis	36/68 (52.9%)
<50%	0/35 (0%)
50–60%	1/35 (2.9%)
60–70%	8/35 (23%)
70–80%	9/35 (25.7%)
80–90%	7/35 (20%)
<90%	10/35 (28.6%)
Aneurysm	17/68 (25%)
Small (<5 mm)	7/16 (43.8%)
Medium (5–10 mm)	9/16 (56.2%)
Large (10–25 mm)	0/16 (0%)
Giant (≥25 mm)	0/16 (0%)
Ruptured	2/16 (12.5%)
AVF	1/68 (1.5%)
Stroke/Emboli	9/68 (13.2%)
TICI <2b	0/7 (0%)
TICI 2c	3/7 (42.9%)
TICI 3	4/7 (57.1%)
Tumor	1/68 (1.5%)
Carotid web	3/68 (4.4%)
Lesion location	
Anterior circulation	54/66 (81.8%)
Posterior circulation	12/66 (18.2%)
Lesion location (artery)	
CCA	1/67 (1.49%)
ICA	26/67 (38.8%)
VA	4/67 (6%)
MCA	15/67 (22.4%)
ECA	0/67 (0%)
Other	21/67 (31.3%)
Previously treated	7/66 (10.6%)
Anatomical tortuosity	
Radial/Brachiocephalic	2/68 (2.9%)
Cervical	0/68 (0%)
ICA	4/68 (5.9%)

*Data are mean ± SD or n (%). SAH, subarachnoid hemorrhage; ICH, intracerebral hemorrhage; AVF, arteriovenous fistula; TICI, thrombolysis in cerebral infarction; CCA, common carotid artery; ICA, internal carotid artery; VA, vertebral artery; MCA, middle cerebral artery; ECA, external carotid artery*.

### Procedure Characteristics

Procedural characteristics are presented in Table 2. Thirty-seven of the 68 total patients underwent stenting treatment (37/68, 54.4%), and the remaining patients were treated with flow diversion (12/68, 17.6%), mechanical thrombectomy (8/68, 11.8%), endovascular coiling (5/68, 7.4%), liquid embolization (3/68, 4.4%), balloon angioplasty (2/68, 2.9%), and balloon angioplasty with stenting (1/68, 1.5%). The Ballast 088 long guiding sheath length most frequently utilized was 90 cm (58/68, 85.3%), followed by 80 cm (6/68, 8.82%), and 100 cm (4/68, 5.9%). The Ballast tip was placed in the ICA (34/67, 50.7%), CCA (16/67, 23.9%), VA (6/67, 9.0%), ECA (2/67, 3.0%), and MCA (1/67, 1.5%) during these endovascular procedures. The target location was reached with technical success in all 68 patients. Median time for puncture to closure was 41 ± 23 min, and median time for both fluoroscopy and radiation exposure was 14.7 ± 13.3 min. Access points included femoral (51/67, 76.1%) and transradial (16/67, 23.9%). Severe radial loops were encountered in two patients (2/68, 2.9%), significant to severe ICA tortuosity was observed in three patients (3/68, 4.4%), and moderate ICA tortuosity was observed in 1 (1/68, 1.5%). Regardless of how acute the angle encountered, we did not experience a single catheter kink in any of our radial cases. Treatment-related procedural complications include mild spasm in the cervical segment of one patient (1/68, 1.5%) with an aneurysm treated with verapamil. Further, the approach was aborted in two patients (2/68, 2.9%) due to complications unrelated to the Ballast long guiding sheath. No Ballast-related complications or adverse events were encountered.

**Table 2:** Procedure characteristics.

**Characteristic**	**Frequency**
General anesthesia	29/67 (43.3%)
Thrombolytics	1/66 (1.52%)
Anticoagulant/Antiplatelet	58/68 (85.3%)
Procedure	
Balloon angioplasty	2/68 (2.94%)
Balloon angioplasty, stenting	1/68 (1.47%)
Endovascular coiling	5/68 (7.35%)
Flow diversion	12/68 (17.6%)
Liquid embolization	3/68 (4.41%)
Mechanical thrombectomy	8/68 (11.8%)
Stenting	37/68 (54.4%)
Target treatment device	
Viatrac	11/68 (16.2%)
Surpass streamline	1/68 (1.47%)
Wingspan	5/68 (7.35%)
Y-stent	1/68 (1.47%)
Pipeline	6/68 (8.82%)
Onyx 18	3/68 (4.41%)
Penumbra	3/68 (4.41%)
XACT	9/68 (13.2%)
WEB	4/68 (5.88%)
ATLAS	1/68 (1.47%)
Resolute Onyx	11/68 (16.2%)
TIGER	1/68 (1.47%)
Gateway	3/68 (4.41%)
Solitaire	2/68 (2.94%)
Trevo	1/68 (1.47%)
Embotrap	1/68 (1.47%)
HydroFrame/HydroSoft	3/68 (4.41%)
Microcoils	1/68 (1.47%)
Optima	1/68 (1.47%)
Catheter position	
CCA	16/67 (23.9%)
ICA	34/67 (50.7%)
VA	6/67 (8.96%)
MCA	1/67 (1.49%)
ECA	2/67 (2.99%)
Other	8/67 (11.9%)
Ballast length (cm)	
80	6/68 (8.82%)
90	58/68 (85.3%)
100	4/68 (5.88%)
Placement of ballast tip	
CCA	16/67 (23.9%)
ICA	34/67 (50.7%)
VA	6/67 (8.96%)
MCA	1/67 (1.49%)
ECA	2/67 (2.99%)
Other	8/67 (11.9%)
Access approach	
Femoral	51/67 (76.1%)
Radial	16/67 (23.9%)
Radiation exposure (min), median ± IQR	14.7 ± 13.3
Fluoroscopy time (min), median ± IQR	14.7 ± 13.3
OR time (min), median ± IQR	110 ± 46.5
Puncture to closure time (min), median ± IQR	41 ± 23

*Data are median ± IQR or n (%). CCA, common carotid artery; ICA, internal carotid artery; VA, vertebral artery; MCA, middle cerebral artery; ECA, external carotid artery*.

### Illustrative Cases

#### Case 1: Stenting of Right Middle Cerebral Artery Stenosis

**Figure 1:**
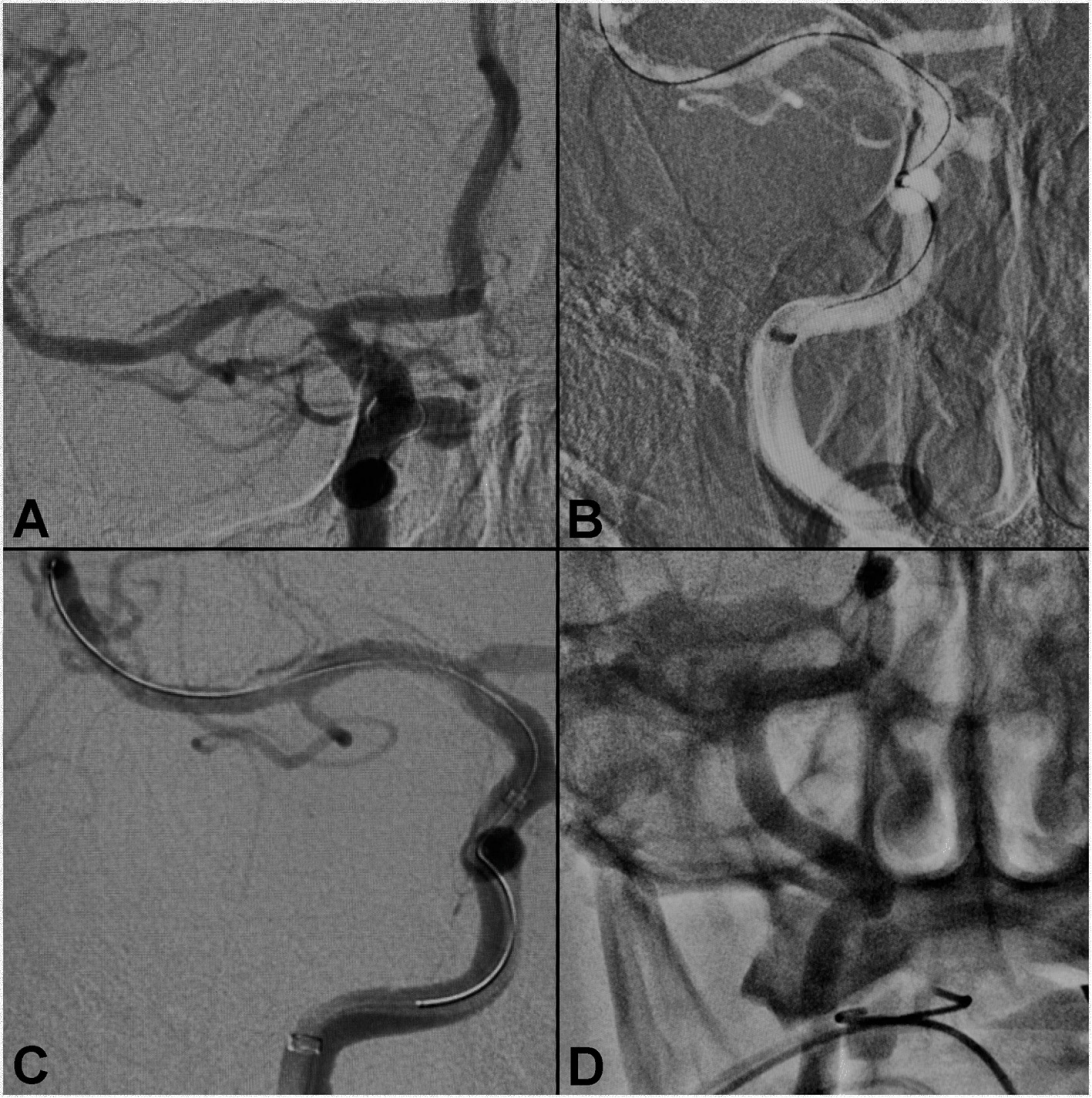
Right middle cerebral artery stenosis. **(A)** Pre-procedure angiogram demonstrating intracranial stenosis of the right M1-MCA segment. **(B)** Ballast position in the ICA petrous segment. **(C)** Post-balloon mounted stent deployment. **(D)** Final angiography showing no evidence of vasospasm or dissection.

A patient presented with a paraclinoid aneurysm treated previously with a flow diverter, with 80–90% intracranial stenosis of the right MCA M1 segment (Figure 1). During the stenting procedure, severe proximal tortuosity was encountered. The Ballast long guiding sheath was placed through the cervical loop and into the horizontal petrous segment to enable better distal support for the intermediate catheter, enabling successful stent deployment. The patient had excellent angiographic results, with complete resolution of the M1 stenosis, with no evidence of dissection or vasospasm.

#### Case 2: Right Carotid Stenting—Radial Approach

**Figure 2:**
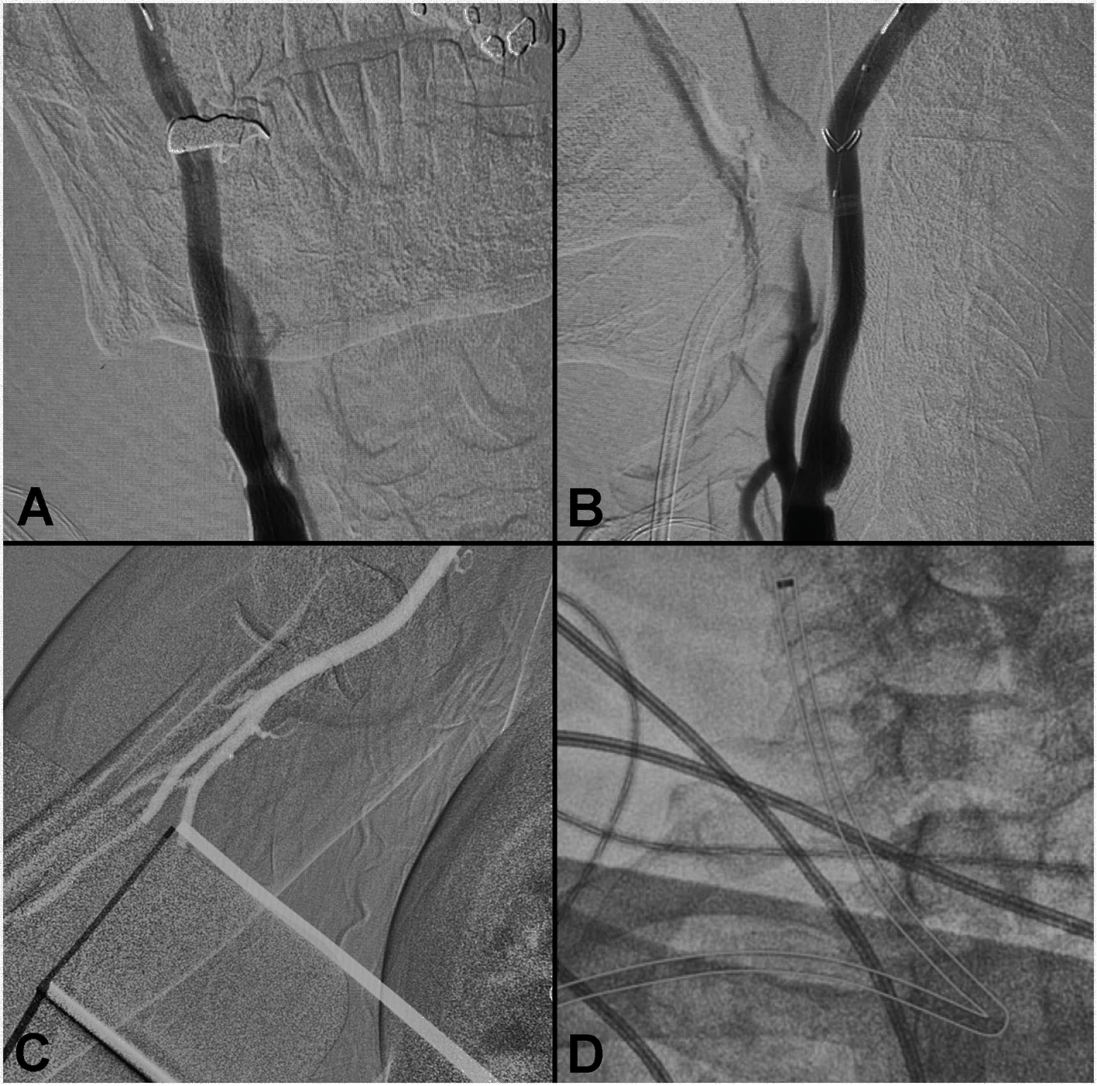
Right carotid stenting—radial approach. **(A,B)** Pre-procedure angiogram demonstrating a right carotid web. **(C)** Radial artery. **(D)** Ballast position post-stenting (outlined).

A patient presented with recurrent transient ischemic attacks and several comorbidities. Non-invasive imaging identified stenosis of the right carotid bulb, and cerebral angiogram confirmed the presence of a right carotid web (Figure 2). Carotid stenting via a right radial approach was performed successfully. The Ballast long guiding sheath was able to navigate the difficult acute angle from the subclavian to the right common carotid without kinking or ovalization, and without losing position during stent delivery.

#### Case 3: Balloon-Assisted Coiling of a Posterior Communicating Artery Aneurysm

**Figure 3:**
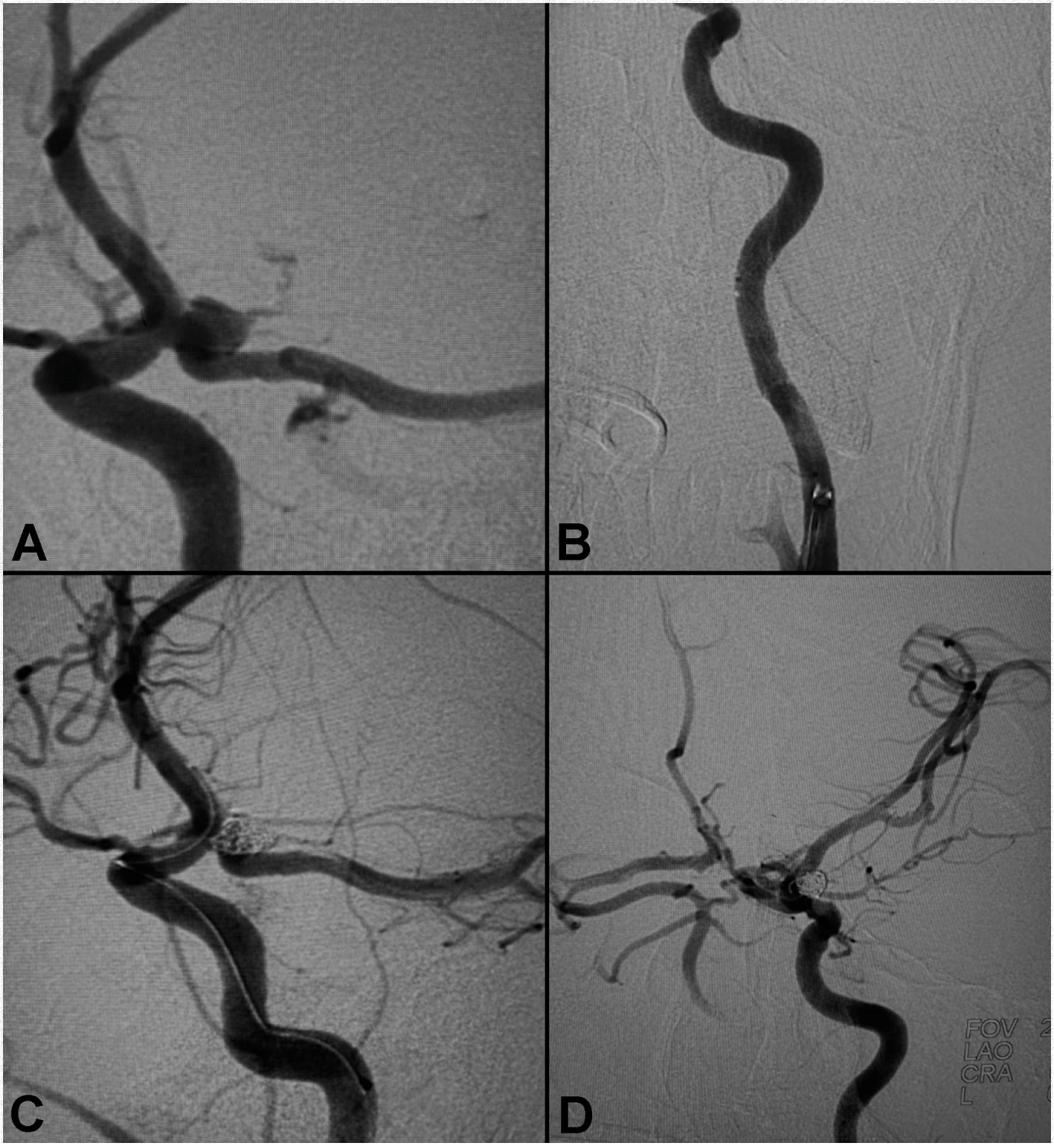
Balloon-assisted coiling of a posterior communicating artery aneurysm. **(A)** Pre-procedure angiogram demonstrating a large PCA aneurysm. **(B)** Proximal anatomy. **(C)** Peri-coiling. **(D)** Post-coiling angiogram demonstrating no residual filling.

A patient presented to the ER with complaints of headache, altered mental status, right sided weakness, altered coordination, and respiratory distress. The patient developed nausea and a headache that intensified over time, and was unresponsive upon arrival to the ER. CT showed SAH, a right midline shift, and a large left temporal lobe hematoma measuring 4.8 × 5.5 cm, with a volume of roughly 66 ml (Figure 3). The patient was transferred to Valley Baptist for endovascular treatment with Hunt & Hess 3 and Fisher Grade 4 scores. Cerebral angiogram demonstrated a left fetal PCA aneurysm measuring 5.3 × 4.6 × 3.8 mm, with a 3.2 mm neck. Balloon assisted coiling with a left side of access was performed to preserve the ostium of the posterior communicating artery (PCOM). A Ballast.088 long guiding sheath was placed in the proximal cervical segment, and its large ID enabled introduction of both the balloon and coiling catheter. Post-interventional visualization demonstrated no significant residual filling. The patient required a decompressive left frontal craniectomy and placement of an EVD, which was removed after 13 days.

#### Case 4: Flow Diverter Deployment for Right Middle Cerebral Artery Aneurysm

**Figure 4:**
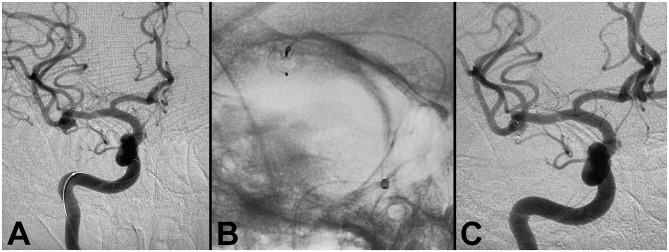
Flow diverter deployment for right middle cerebral artery aneurysm. **(A)** Pre-procedure angiogram demonstrating right MCA aneurysm. **(B)** WEB flow diverter deployment, demonstrating successful deployment with conformity to the aneurysm walls. **(C)** Post-procedure angiogram demonstrating successful occlusion.

A patient was referred for evaluation of a MCA aneurysm diagnosed on CT angiography after presenting with neck pain from a fall. A diagnostic angiogram confirmed the presence of a right MCA bifurcation aneurysm measuring 6.1 × 5.5 × 4.4 mm, with a 3.5 mm neck (Figure 4). Treatment with a 6 × 3 mm single layer intrasaccular device was performed. Mild tortuosity of the ICA required good proximal support for the 0.021” delivery microcatheter. The Ballast 0.088 long guiding sheath enabled distal navigation in this case to the horizontal petrous segment. Successful deployment with good conformity to the aneurysm walls resulted in excellent angiographic outcomes.

#### Case 5: Aspiration Thrombectomy for Right Middle Cerebral Artery Occlusion

**Figure 5:**
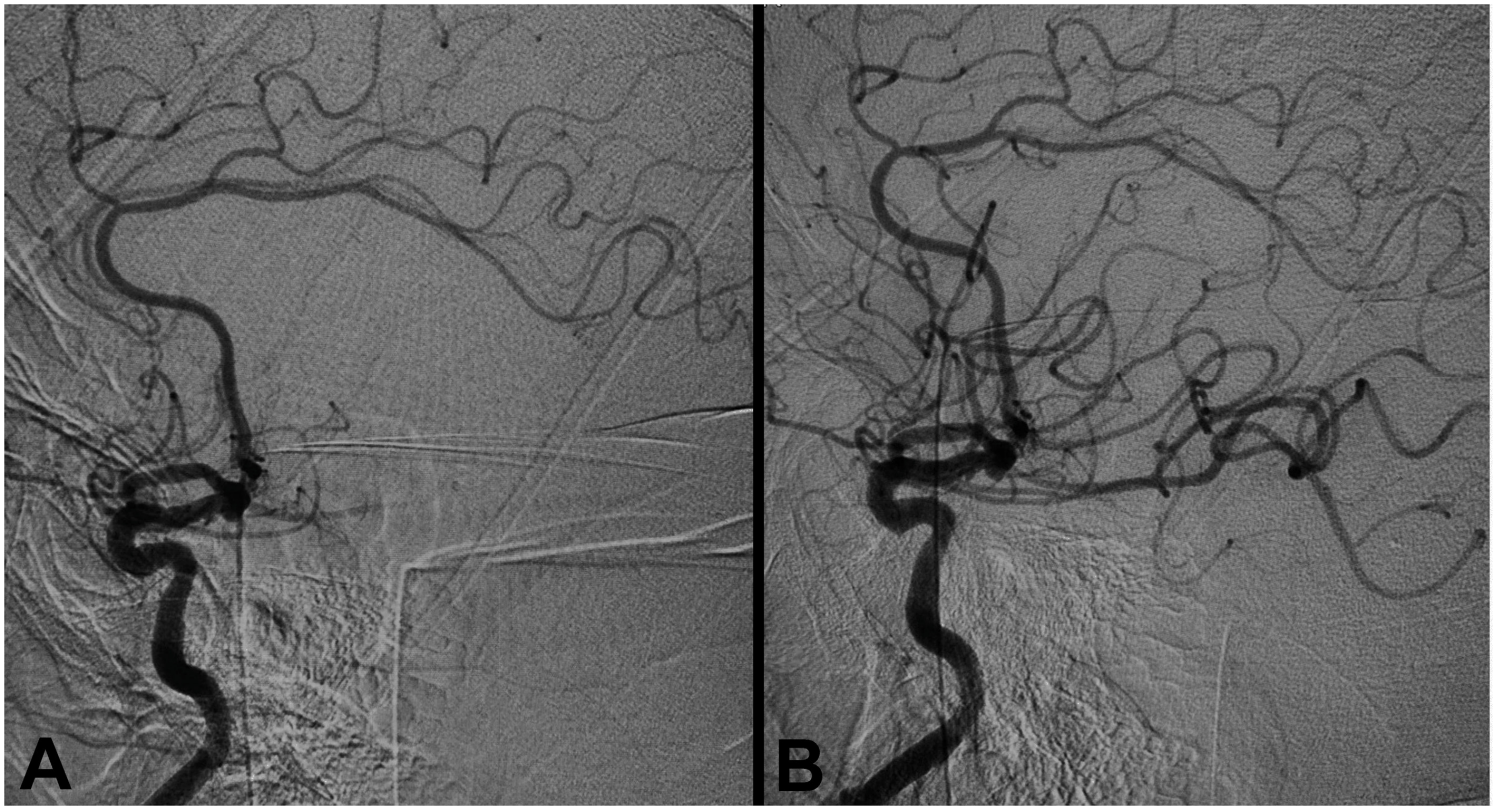
Aspiration thrombectomy for right middle cerebral artery occlusion. **(A)** Pre-procedure angiogram demonstrating occlusion of the right M1 segment (TICI 0). **(B)** Post-thrombectomy angiogram demonstrating TICI 3 reperfusion.

A patient presented with left side weakness, slurred speech, and a NIHSS of 18. The patient was on rivaroxaban for atrial fibrillation, and had a history of hypertension and hyperlipidemia. Due to use of anticoagulants, IV-tTPA was not administered, and the patient was transferred to Valley Baptist Medical Center for further treatment. On arrival, CT perfusion showed a right MCA penumbra, while CT angiography demonstrated decreased flow to the right MCA branches (Figure 5). The patient was treated with mechanical thrombectomy for complete occlusion of the right M1. Access was obtained with the Ballast 0.088 long guiding sheath, which provided excellent arch support for delivery of a large bore 0.072 aspiration catheter. Complete recanalization and thrombolysis in cerebral infarction (TICI) 3 reperfusion was achieved.

## Discussion

In this report, we describe our experience with the Ballast.088 long guiding sheath to deliver treatment devices in a series of patients with cerebrovascular disease, including intracranial and extracranial stenosis, aneurysm, stroke, AVF, and carotid web. Ballast can be used for direct access to the cerebral vasculature or through a short vessel access sheath. In the present study, the Ballast long guiding sheath was navigated to the desired anatomical locations in the anterior or posterior circulation successfully using both transradial and transfemoral access in a range of patient anatomies. Compared to our experience with predicate guide catheter systems, we found the Ballast long guiding sheath to offer significantly improved support and trackability in both the radial and femoral cases, navigating higher without increasing the risk of dissection. Overall, Ballast provided support for the delivery of treatment devices to a range of target locations with technical success and with no Ballast-related complications.

The development of flow diversion devices for the next generation of neuroendovascular therapeutic techniques prompted a paradigm shift from biaxial to triaxial delivery systems; as flow diverters such as the PED have large self-expanded diameters of up to 5.25 mm, the IDs of delivery microcatheter increased accordingly (14–16). In parallel, the rise of aspiration utilization in acute ischemic stroke (17, 18) has led to the development of hyperflexible, large-bore DACs for enhanced trackability (15, 19, 20). However, the hyperflexibility of these catheters means that they depend on the guide catheter or sheath for proximal shaft support (20). When used in conjunction with long guiding sheaths, such as the Ballast, these DACs can provide the proximal support needed while simultaneously maintaining flexibility for improved navigation. This system allows for the delivery of a wide range of therapeutic devices through a larger ID, which is crucial for large modern neuroendovascular devices, such as flow diverters (15, 21).

Several triaxial catheter systems with long guiding sheaths have been used with success in endovascular procedures. One of the earliest models of long guiding sheaths developed is the Flexor Shuttle Sheath (Cook Medical), which is reinforced with coil, has a flat distal tip, and was initially designed for carotid artery stenting (22). The Shuttle has been used for endovascular treatment in multiple prospective and retrospective studies since its early inception (7, 15, 23–25). In 2002, Ohki et al. describe their initial experience using the Shuttle in 31 total patients for carotid artery stenting (26). Technical success was achieved in 97% of patients, with a stroke unrelated to the catheter itself occurring in 1 (1/31, 3.33%) patient. The Shuttle has since been used flow diversion (25), manual aspiration thrombectomy (24), and control angiography (27), with similar rates of success. Despite propelling a new age of long guiding sheaths, the Shuttle's introducer sheath tip is stiff, and the sheath itself is less flexible than most guiding catheters, challenging navigation of the Shuttle through tortuous vessels and vertebral arteries (27). These limitations may lead to complications such as vasospasm or dissection during deployment of flow diverters or DACs. For these reasons, some neurointerventionalists do not routinely use the Shuttle for tortuous aortic anatomies (27).

Additional long guiding sheaths include the Neuron Max (Penumbra) and AXS Infinity (Stryker). These advanced catheter systems have also been widely used to deliver treatment devices in diverse neuroendovascular procedures in recent years. Many studies have been published using a triaxial system with the Neuron Max long guiding sheath in a number of endovascular procedures with good outcomes (24, 28–30). More recently, the AXS Infinity also achieved high rates of technical success and few catheter-related complications (3, 15, 22). Lin et al. describe their experience with the AXS Infinity to deliver PED Flex treatment to 95 patients with cerebral aneurysms (22). They reported few catheter access-related complications, including asymptomatic iatrogenic dissection in one case (1/95, 1.1%) and groin hematoma in one case (1/95, 1.1%) (22). Their findings demonstrate the utility of long guiding sheaths in the augmentation of neurointerventional procedures with a triaxial system.

Both the Neuron Max and AXS Infinity offer improvements to the catheter design compared to the earlier Flexor Shuttle Sheath. Modifications including stainless steel braided shaft reinforcement, larger outer and IDs, multiple transition zones, and in the AXS Infinity, a round distal tip. Similar to Neuron Max and AXS Infinity, Ballast is braid-reinforced with a single lumen, variable stiffness, and a radiopaque zone at the distal end; however, Ballast also has a luer hub on the proximal end, a progressively softer coiled distal tip, and extended hydrophilic coating to both improve navigation and reduce friction. We believe that one of the most import attributes of novel supportive guide catheters, like Ballast, is to be soft enough to track around cervical loops and access petrous segments without dissection. The Ballast sheath's 0.100 inch distal outer diameter (OD) is smaller than predicate long guiding sheaths, enhancing the atraumatic navigation of Ballast through the tortuous neurovasculature. Furthermore, its large proximal ID of 0.088 inch ensures compatibility with a wide range of currently available endovascular devices. With functional lengths ranging from 80 to 105 cm, this long guiding sheath is optimally designed to accommodate distal anatomies. Together, the key innovations of this system provide the perfect balance between proximal support and distal softness needed to achieve improved trackability, smooth navigation, and kink resistance. A recent retrospective analysis comparing NeuronMax and Ballast performance during thrombectomy found shorter procedure times and faster puncture to access time with Ballast (31). Rates of successful revascularization and good 90-day outcomes were similar for both catheters (31). In our experience, the design advancements with Ballast enable the safe delivery of treatment devices in an atraumatic manner without evidence of complications attributable to its positioning.

## Limitations

The lack of a comparison group of Ballast against other guide technologies used at Valley Baptist Medical Center for neuroendovascular procedures is a limitation of this study. Furthermore, our analysis is limited to retrospective, rather than prospective data from a single medical center making our results less generalizable. Although our small sample size was also small in comparison to randomized multicenter trials, our experience with a relatively new long guiding sheath device demonstrates the early clinical efficacy of the Ballast long guiding sheath. Additional analyses should be conducted to compare the outcomes of Ballast to the other existing models of long guiding sheaths.

## Conclusion

Long guiding sheaths provide crucial support for intracranial catheters through distal circulation, enabling the successful delivery of modern neuroendovascular treatment devices for a range of disease states. Selecting the appropriate distal access guiding systems for endovascular intervention requires sufficient navigability without sacrificing supportability for working devices. The design characteristics for the Ballast long guiding sheath are tailored specifically to enhance treatment within the distal vasculature, with variable stiffness, optimal lubricity, and a small distal OD to improve pushability, trackability, proximal support. We demonstrate the feasibility of using the Ballast to successfully deliver modern neurointerventional treatment devices to desired locations for a variety of intracranial conditions.

## Data Availability Statement

The original contributions presented in the study are included in the article/supplementary material, further inquiries can be directed to the corresponding author/s.

## Ethics Statement

The studies involving human participants were reviewed and approved by the MetroWest institutional review board. The patients provided their written informed consent to participate in this study.

## Author Contributions

AH, WT, and MM contributed to study design, data acquisition and interpretation, and critically revised the article for scientific content. EB contributed to data analysis, manuscript preparation, and critical revisions. All authors gave final approval of the version to be published.

## Conflict of Interest

AH serves as a consultant and speaker for GE Healthcare, Medtronic, Stryker, Microvention, Penumbra, Balt USA, and Genentech. EB contracts with Superior Medical Experts. The remaining authors declare that the research was conducted in the absence of any commercial or financial relationships that could be construed as a potential conflict of interest.
